# Ampullary gangliocytic paraganglioma with lymph node metastasis: A case report with literature review

**DOI:** 10.1097/MD.0000000000029138

**Published:** 2022-04-15

**Authors:** Hanlim Choi, Jae-Woon Choi, Dong Hee Ryu, Sungmin Park, Myung Jo Kim, Kwon Cheol Yoo, Chang Gok Woo

**Affiliations:** a Department of Surgery, Chungbuk National University College of Medicine, Cheongju, Korea; b Department of Surgery, Chungbuk National University Hospital, Cheongju, Korea; c Department of Pathology, Chungbuk National University Hospital, Cheongju, Korea.

**Keywords:** ampulla, case report, gangliocytic paraganglioma, lymph node, metastasis

## Abstract

**Rationale:**

Gangliocytic paraganglioma (GP) is a rare tumor that mostly develops in the duodenum and is composed of the following 3 cell types: epithelioid endocrine, spindle-like, and ganglion-like cells. It manifests as symptoms such as abdominal pain, gastrointestinal bleeding, and weight loss; however, occasionally, it is incidentally detected on endoscopic or radiologic examinations. Although GP is usually benign, it can metastasize to the lymph nodes, and distant metastases have been reported in some cases.

**Patient concerns:**

A 46-year-old woman presented with anemia on health surveillance examination. She had no other specific symptoms, and her physical examination did not reveal any abnormal finding.

**Diagnosis:**

Endoscopic ultrasound-guided fine-needle aspiration biopsy was performed, and the endoscopist obtained samples from the inner side of the ampullary mass. Pathological examination suggested GP or a neuroendocrine tumor.

**Interventions:**

Initially, we planned transduodenal ampullectomy with lymph node excision. However, there was severe fibrosis around the duodenum, and an examination of a frozen biopsy sample from the periduodenal lymph node showed atypical cells in the lymph node. Therefore, we performed pylorus-preserving pancreaticoduodenectomy with lymph node dissection.

**Outcomes:**

The final pathological diagnosis was GP located in the ampulla of Vater. The GP showed lymphovascular and perineural invasion and invaded the duodenal wall. Furthermore, 4 out of 18 harvested lymph nodes showed metastasis.

**Lessons:**

We described a case of GP confined to the ampulla with regional lymph node metastasis and reviewed published literature on ampullary GP with lymph node metastasis.

## 1 Introduction

Gangliocytic paraganglioma (GP) is a rare tumor that typically develops in the duodenum; it is composed of 3 types of cells: epithelioid endocrine, spindle-like, and ganglion-like cells.^[1]^ It manifests as symptoms including abdominal pain, gastrointestinal bleeding, and weight loss. Nevertheless, occasionally, GP is detected on endoscopic or radiologic examination incidentally.^[2]^ Although GP is generally benign, metastasis to the lymph nodes could develop, and distant metastases have been reported in some cases.^[3–5]^ Here, we describe a case of GP confined to the ampulla with regional lymph node metastasis and review the published literature on ampullary GP with lymph node metastasis.

## 2 Case presentation

**Figure 1:**
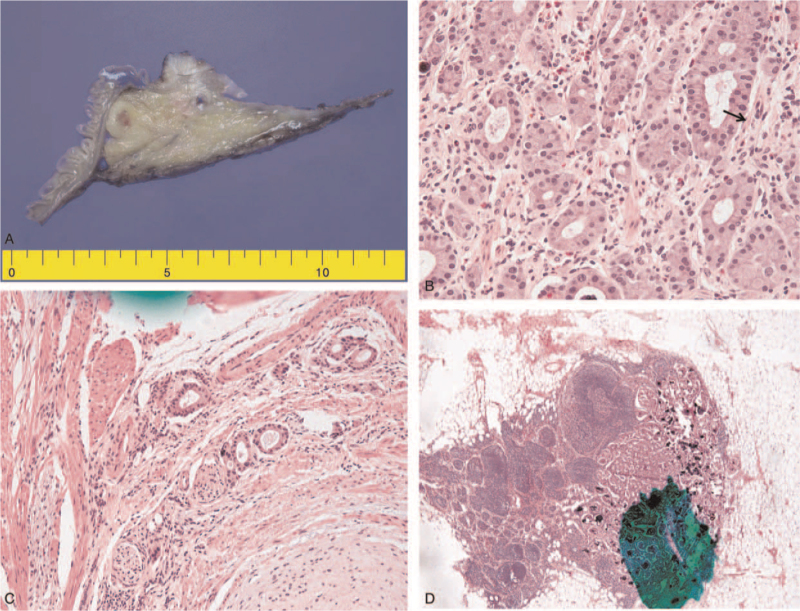
A. Grossly, A well demarcated mass located in ampulla of Vater. B. Nesting and glandular arrangement of epithelioid cells (hematoxylin and eosin [HE] staining, ×400). C. Perineural invasion (HE staining, ×400). D. Lymph nodes showing metastasis (HE staining, ×400).

A 46-year-old woman presented with anemia on her health surveillance examination. She had no other specific symptoms, and no underlying disease. Although her physical examination was normal, laboratory test revealed her hemoglobin and hematocrit levels to be 7.7 g/dL and 34.5%, respectively. Her carcinoembryonic antigen and carbohydrate antigen 19-9 levels were normal. Initial esophagogastroduodenoscopy showed a firm, bulging fibrotic ampullary mass with diffuse edema. Subsequent endoscopic ultrasound revealed a mass (1.5 cm × 0.8 cm) with a periduodenal lymph node (1.2 cm × 0.8 cm). The first fine-needle aspiration biopsy revealed inflammation. In the second endoscopic ultrasound-guided fine-needle aspiration biopsy, the endoscopist obtained a sample from the inner side of the ampullary mass. Pathological examination suggested GP or a neuroendocrine tumor. Abdominopelvic computed tomography revealed a slightly more prominent nodular enhanced lesion with diffusion restriction at the ampulla of Vater with dilatation of the biliary tree and pancreatic duct. Because the mass was sessile with lymph node enlargement, we planned transduodenal ampullectomy with lymph node excision. During the operation, severe fibrosis was noted around the duodenum, and a frozen biopsy of the periduodenal lymph node showed atypical cells in the lymph node. Thereafter, we performed pylorus-preserving pancreaticoduodenectomy (PD) with lymph node dissection. Histological examination revealed the tumor to be unencapsulated and located in the ampulla; epithelioid cells were detected in the tumor (Fig. 1A and B).

**Figure 2:**
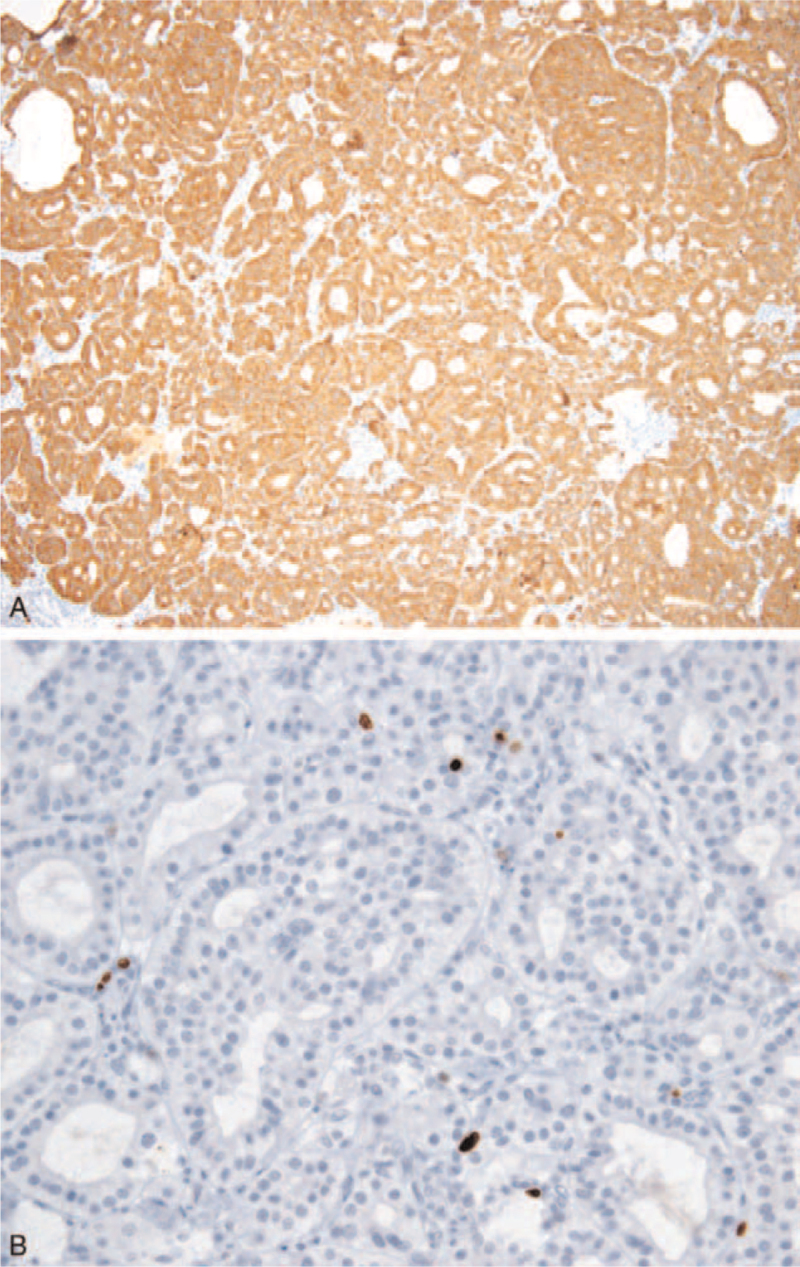
Immunohistochemistry. A. Tumor showing cytoplasmic positivity for synaptophysin in epithelioid cells (×200). B. Tumor showing less than 1% Ki-67 labeling index (×400).

The final pathological examination showed GP located in the ampulla of Vater and the size of the tumor was approximately 2.4 cm. The tumor showed lymphovascular and perineural invasion and it invaded the duodenal wall (Fig. 1C). Four of 18 harvested lymph nodes showed metastasis (Fig. 1D). Immunohistochemistry revealed the tumor was positive for S-100, synaptophysin, chromogranin A, and neuron-specific enolase. Furthermore, Ki-67 proliferative activity was lower than 1% (Fig. 2A and B). The patient was discharged on the 11th day after the operation. She did not receive any adjuvant treatment and showed no recurrence or metastasis 3 years after the operation.

## 3 Discussion and conclusions

After duodenal ganglioneuroma was first reported by Dahl et al^[6]^ in 1957, GP was named by Kepes and Zacharias^[7]^ in 1971. In a review by Okubo et al^[8]^ published in 2011, in 90% of the cases, GP developed in the duodenum and the average tumor size was 2.5 cm. Although the common symptoms of GP include abdominal pain, gastrointestinal bleeding, anemia, and weight loss, occasionally, GP has been found incidentally.^[9]^ GP has a characteristic histological appearance, including being composed of epithelioid, spindle, and ganglion cells. A definitive diagnosis of GP before surgery could be challenging. In the case of our patient, GP was detected by preoperative endoscopic biopsy and confirmed by postoperative pathological examination.

Although reports of local lymph node involvement in GP are rare, based on past research, the actual incidence can be 10% to 20%.^[10]^ Of the cases of GP reported hitherto, we searched for cases in which GP was localized to the ampulla and metastasized to the lymph node, and found 12 cases that were described in English (Table 1). Among these 12 cases, in 1 case, the patient underwent only local resection and there were no signs of recurrence for 20 months.^[11]^ Two other patients underwent local resection followed by PD or pylorus-preserving PD. In 1 case, massive hematemesis prompted an exploratory laparotomy and local resection of the tumor.^[12]^ In another case, PD was performed after local resection because of positive resection margins.^[15]^ Follow-up surveillance revealed no metastatic disease/lymph node involvement. Tumor spread through the submucosal layer was associated with a higher rate of lymph node spread and was seen more frequently in women. It is hypothesized that sex hormones may have some relationship with tumor extension.^[8]^

**Table 1:** Summary of reported patients having ampullary gangliocytic paraganglioma with lymph node metastasis.

Reference	Age (yr)	Sex	Presenting symptoms	Largest diameter (mm)	Positive LN (n)	Surgery	Recurrence-free survival (mo)
Büchler et al^[11]^	50	M	GI bleeding	30	1	Local resection	20
Inai et al^[12]^	17	M	GI bleeding	20	1	Local resection followed by PD^∗^	32
Hashimoto et al^[13]^	47	M	Asymptomatic	65	1	PD	14
Bucher et al^[14]^	31	F	GI bleeding	30	1	PPPD	44
Witkiewicz et al^[15]^	38	F	Abdominal pain	15	2	Local resection followed by PPPD^†^	NR
Ghassemi et al^[16]^	62	F	GI bleeding	NR	1	PPPD^†^	12
Okubo et al^[3]^	61	M	GI bleeding	30	1	PPPD	6
Ogata et al^[17]^	16	M	GI bleeding	35	4	PPPD	36
Barret et al^[18]^	51	F	GI bleeding	25	2	PD	96
Shi et al^[19]^	47	M	Abdominal pain	40	8	PD	24
Dowden et al^[2]^	59	F	Abdominal pain	28	2	PPPD	5
Mishra et al^[20]^	50	F	Abdominal pain	18	2	PD	6
Current case	46	F	Anemia	24	4	PPPD	48

F = female, GI = gastrointestinal, LN = lymph node, M = male, NR = no records, PD = pancreaticoduodenectomy, PPPD = pylorus-preserving pancreaticoduodenectomy.

∗ Emergent local resection followed by PD.

† Local resection followed by PD because of a positive resection margin.

There are also reports describing the association between GP and lymph node metastasis. These reports suggest that epithelial components have a high malignant potential, because only epithelioid elements have been observed in most lymph node metastases.^[11,12]^

Regarding treatment, in cases in which the tumor is confined to the ampulla and there is no regional lymph node spread, endoscopic mucosal resection and ampullary resection are both viable options.^[10]^ However, in the case of local or lymphovascular infiltration, more aggressive surgery should be performed, such as PD.^[21]^ Although GP with lymph node metastasis is rare, local lymph nodes should be excised during surgical treatment.

## Author contributions

All authors participated in the management of the patient described in this case report. HC collected all the references and was a major contributor in the writing of the manuscript. All authors have read and approved the manuscript.

**Conceptualization:** Hanlim Choi, Jae-Woon Choi, Chang Gok Woo.

**Data curation:** Hanlim Choi, Myung Jo Kim, Chang Gok Woo.

**Formal analysis:** Hanlim Choi, Sungmin Park.

**Investigation:** Hanlim Choi, Dong Hee Ryu, Sungmin Park, Myung Jo Kim, Chang Gok Woo.

**Methodology:** Myung Jo Kim.

**Resources:** Kwon Cheol Yoo.

**Supervision:** Jae-Woon Choi, Dong Hee Ryu.

**Validation:** Dong Hee Ryu.

**Writing – original draft:** Hanlim Choi.

**Writing – review & editing:** Hanlim Choi, Jae-Woon Choi, Dong Hee Ryu, Sungmin Park, Kwon Cheol Yoo, Chang Gok Woo.
